# Differential degeneration of the ACTAGT sequence among *Salmonella*: a reflection of distinct nucleotide amelioration patterns during bacterial divergence

**DOI:** 10.1038/s41598-017-11226-9

**Published:** 2017-09-08

**Authors:** Le Tang, Emilio Mastriani, Yu-Jie Zhou, Songling Zhu, Xin Fang, Yang-Peng Liu, Wei-Qiao Liu, Yong-Guo Li, Randal N. Johnston, Zheng Guo, Gui-Rong Liu, Shu-Lin Liu

**Affiliations:** 1 Systemomics Center, College of Pharmacy, and Genomics Research Center (State-Province Key Laboratories of Biomedicine-Pharmaceutics of China), Harbin Medical University, Harbin, China; 2 HMU-UCFM Centre for Infection and Genomics, Harbin Medical University, Harbin, China; 3 Department of Ecosystems and Public Health, University of Calgary, Calgary, Canada; 4 Translational Medicine Research and Cooperation Center of Northern China, Heilongjiang Academy of Medical Sciences, Harbin, China; 5 Department of Microbiology, Immunology and Infectious Diseases, University of Calgary, Calgary, Canada; 6 Department of Infectious Diseases of First Affiliated Hospital, Harbin Medical University, Harbin, China; 7 Department of Biochemistry and Molecular Biology, University of Calgary, Calgary, Canada; 8 College of Bioinformatics Science and Technology, Harbin Medical University, Harbin, China; 9 Present Address: Division of Neurology, Department of Medicine, University of British Columbia, Vancouver, Canada; 10 Present Address: Department of Clinical Neurosciences, University of Calgary, Calgary, Canada

## Abstract

When bacteria diverge, they need to adapt to the new environments, such as new hosts or different tissues of the same host, by accumulating beneficial genomic variations, but a general scenario is unknown due to the lack of appropriate methods. Here we profiled the ACTAGT sequence and its degenerated forms (i.e., hexa-nucleotide sequences with one of the six nucleotides different from ACTAGT) in *Salmonella* to estimate the nucleotide amelioration processes of bacterial genomes. ACTAGT was mostly located in coding sequences but was also found in several intergenic regions, with its degenerated forms widely scattered throughout the bacterial genomes. We speculated that the distribution of ACTAGT and its degenerated forms might be lineage-specific as a consequence of different selection pressures imposed on ACTAGT at different genomic locations (in genes or intergenic regions) among different *Salmonella* lineages. To validate this speculation, we modelled the secondary structures of the ACTAGT-containing sequences conserved across *Salmonella* and many other enteric bacteria. Compared to ACTAGT at conserved regions, the degenerated forms were distributed throughout the bacterial genomes, with the degeneration patterns being highly similar among bacteria of the same phylogenetic lineage but radically different across different lineages. This finding demonstrates biased amelioration under distinct selection pressures among the bacteria and provides insights into genomic evolution during bacterial divergence.

## Introduction

Bacteria constantly accumulate genomic changes to become increasingly better fit to the changing environment, such as in acquiring new capabilities to exploit nature for available resources, including laterally transferred DNA (LTD, e.g., prophages, genomic islands, etc.) and nucleotide variations (NVs, e.g., base substitutions, short insertion or deletions, etc.). The acquisition of LTD is mostly acute, occasionally bringing in beneficial traits desperately needed by the adapting bacteria, followed in many cases by genomic rebalancing for structural adjustment of the genome^1–3^, and the accumulation of NVs is usually chronic, gradually making sophisticated refinement of the genome in response to the specific selection pressures. Although much has been documented on LTD in bacteria over the past couple of decades due especially to the availability of whole genome sequences for analysis^4–6^, little is known about the general NV processes or patterns. In fact, in contrast to the stochastic nature of LTD acquisition, it is the distinct NVs that would reflect the longitudinal genomic divergence processes in bacterial adaptation. As such, profiling and analysis of NVs would provide unprecedented opportunities to uncover the genetic basis for bacterial evolution from common ancestors to distinct biological, and especially pathogenic, lineages.

Genomic comparisons between closely related bacteria have revealed a broad range of NVs, including mostly nucleotide substitutions and rarely short insertions or deletions of oligonucleotides, throughout the genomes as exemplified by the comparisons between the genetically very closely related but pathogenically highly different pathogens *S. paratyphi* C and *S. choleraesuis* ^7^. The documented NVs are diverse and presumably contain enormous amounts of information regarding bacterial divergence and, especially, pathogenic evolution. However, novel and effective approaches are needed to systematically analyze NVs and reveal general patterns of nucleotide divergence between biologically different bacterial relatives. To look into this issue, we have recently profiled evolutionarily conserved short genomic sequences and inspected their degeneration patterns among closely related bacteria. The SpeI cleavage sequence ACTAGT is one of such conserved short sequences and is rare in enteric bacteria, such as *Escherichia coli* and *Salmonella*, due to the scarcity of the tetranucleotide sequence CTAG that it contains^8, 9^.

The CTAG sequence tends to be eliminated by the Very Short Patch repair system^10–13^. Therefore, all endonucleases that have their cleavage sequences containing CTAG have rare sites in the genomes of *Salmonella*, including BlnI (CCTAGG), which recognizes the same cleavage sequence as AvrII^14^, XbaI (TCTAGA), and NheI (GCTAGC), in addition to SpeI. Whereas the XbaI cleavage sequence patterns, profiled by either bioinformatics^15^ or pulsed field gel electrophoresis (PFGE) analysis^16, 17^, are lineage-specific among *Salmonella* as a result of independent divergence of the ancestral CTAG sequences, information provided by XbaI profiling is limited due to the facts that several XbaI cleavage sites are normally methylated and so are not cleavable by the XbaI enzyme^17^ and that several cleavage sites are located within *rrn* operons and so are often translocated or even removed when the genome is rearranged by recombination between *rrn* genes^2, 3, 18, 19^. Therefore, when some of the XbaI cleavage sites are methylated, moved or removed, analysis by PFGE will be confused. BlnI or AvrII is also problematic in this regard, because they have even more cleavage sites than XbaI in the *rrn* operons^14^. NheI has another problem: too many cleavage sites in the *Salmonella* genomes for the PFGE results to be easily analyzable. On the other hand, the SpeI cleavage sequence seemed to be more suitable than the other three endonucleases for such studies, because it has the appropriate numbers of cleavage sites for PFGE analysis in *Salmonella* and the special advantage of its absence in the *rrn* operons. Previous investigations have shown that the SpeI cleavage patterns are distinct among different *Salmonella* serotypes^8, 18, 20, 20^ and reflect the genomic divergence among the *Salmonella* lineages.

In this study, we profiled the SpeI cleavage sequence ACTAGT and its degenerated forms in the genome of representative *Salmonella* lineages to obtain a snapshot of the genome optimization processes through NV during bacterial evolution. Here we adopt the traditional *Salmonella* classification and nomenclature system^22^ and define a *Salmonella* lineage as a monophyletic *Salmonella* pathogen^23^, such as *S. typhi* (infecting only humans and causing typhoid fever) or *S. typhimurium* (infecting a broad range of hosts and causing gastroenteritis in humans). We found that ACTAGT had much lower frequencies than random appearance, consistent with previous findings, and that the existing ACTAGT sequence is highly conserved across the *Salmonella* lineages or even between *Salmonella* and *E. coli*. Of great significance, many ACTAGT sequences are located in the intergenic regions, suggesting their biological importance. Computer modeling demonstrated stable stem-loop structures in some intergenic regions involving ACTAGT. A very special finding is that some degenerated forms of ACTAGT were also highly conserved, pointing to an adaptive nucleotide substitution event, in which an alternative nucleotide replaces one of the six nucleotides ACTAGT, maintaining the function and in the meantime minimizing the number of the CTAG sequence in the genome, toward an optimal genome construction.

## Methods

### Bacterial strains and genomes

Information on the bacterial strains used in this study can be found at the *Salmonella* Genetic Stock Center (http://www.ucalgary.ca/~kesander/), and sequences of the analyzed genomes were downloaded from NCBI (www.ncbi.nlm.nih.org/genome). We use the traditional *Salmonella* nomenclature^22^ for reasons detailed in a previous publication^23^.

### Phylogenetic analysis

We determined orthologues between the bacteria by BLAST alignment with the criteria that identity was larger than 70% and alignment length was longer than 70% of the whole gene. We then concatenated the conserved genes using home-made Perl scripts to generate the dendrogram. The phylogenetic tree was constructed by MEGA6 with 1000 bootstrap replicates.

### Computer modeling of the short sequence secondary structures

Using the Free Energy Minimization method^24–26^, we conducted the modeling based on the assumption that the total free energy of a given secondary molecular structure is the sum of independent contributions of adjacent or stacked base pairs in stems. For modeling the stem-loop structures, we employed the Vienna RNA Package (http://www.tbi.univie.ac.at/RNA/), which consists of a C code library and several programs including RNAfold. The program reads RNA sequences from stdin, calculates their minimum free energy (mfe) structure and prints to stdout. We used the *mountain.pl* script to produce mountain plots and the Maximum Expected Accuracy method with the program CONTRAfold^27^ to predict the pseudo-knot-free structures. CONTRAfold uses the maximum P (i, j) expected accuracy approach for the predictions. We also used RNAstructure (Mathews Lab, University of Rochester Medical Center, Department of Biochemistry and Biophysics), which contains the MaxExpect program (http://rna.urmc.rochester.edu/RNAstructureWeb), for the analysis. To run the programs for the prediction task, we employed an HPC Cluster based on Ubuntu 14.04.2 LTS (Trusty), Kernel 3.16.0-30-generic, using the Sun Grid Engine 6.2u5-7.3 amd64 as queue manager and scheduler to accept jobs. The programs were run on the cluster in the *“trivial parallel computing”* way and the results were obtained from RNAfold v. 2.19 and MaxExpect v. 5.6 linked to Perl v.5.18.2.

### Modeling the structure

We used two scripts, *mountain.pl* and *relplot.pl*, to predict pair probabilities within the equilibrium ensemble and produce a diagram of the predicted structure containing information about probability, respectively. The Perl script *relplot.pl* adds reliability information to the RNA secondary structure plot and computes a well-definedness measure.

### The MEA approach to predict the secondary structure

In order to be more confident about the obtained results, we also applied the MaxExpect program to execute the following command:

MaxExpect–sequence LT2-SpeI.fasta LT2-SpeI.out–gamma 1–percent 10–structures 20–window

Then a file would be generated with a file name (here LT2-Spe.out, to be specified each time), which contains information about the predicted structure.

### Protein structure modeling

We used the Phyre2 program (http://www.sbg.bio.ic.ac.uk/phyre2/html/page.cgi?id=index) to model the structural changes of proteins deduced from nucleotide sequences.

## Results

### Frequencies of ACTAGT and its degenerated forms in *Salmonella* and *E. coli*

**Figure 1:**
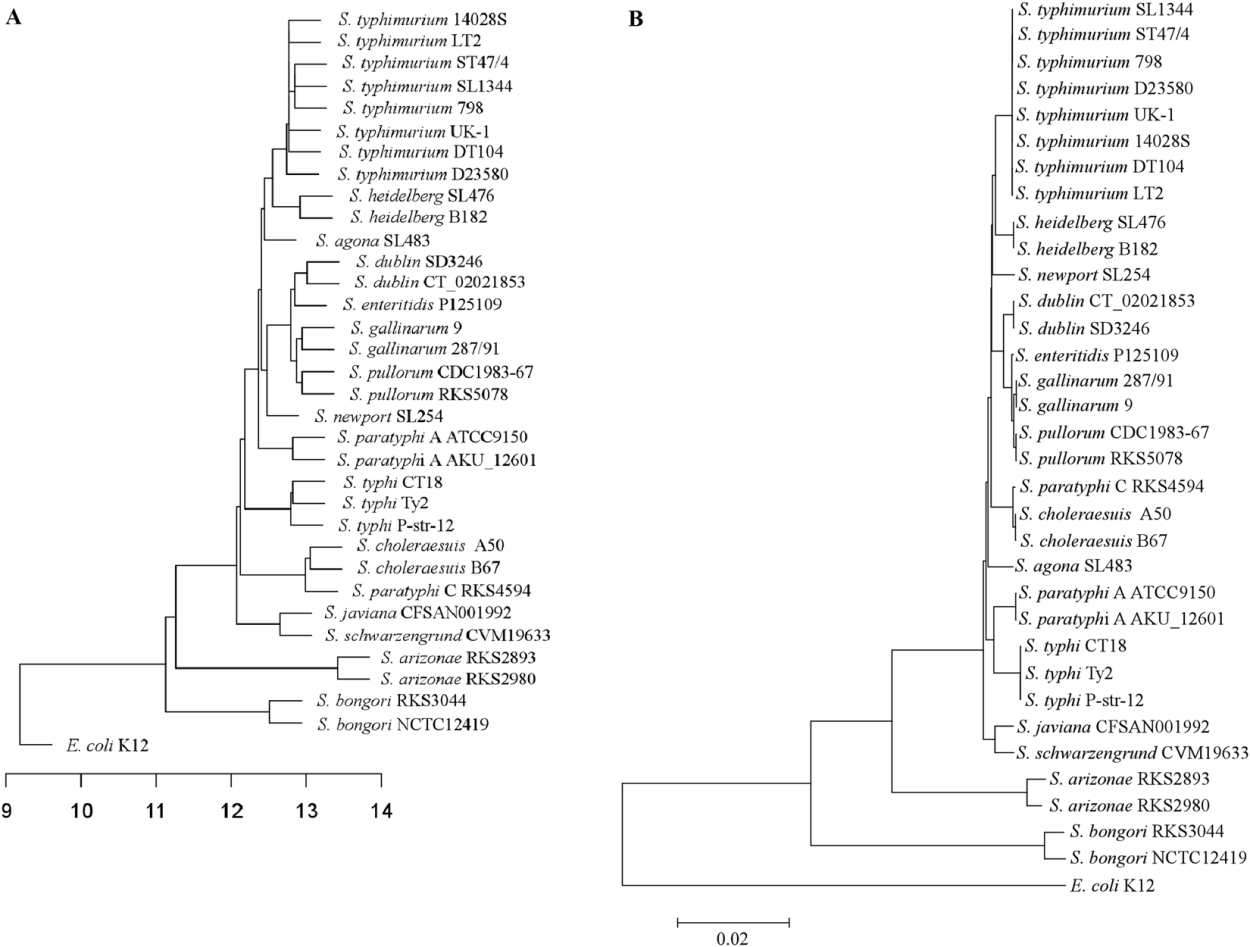
Phylogenetic trees among the *Salmonella* strains and *E. coli* K12 based on the similarity of genomic distribution of the hexanucleotide ACTAGT (**A**) and on core genome (**B**).

We first profiled the ACTAGT sequences in *S. typhimurium* LT2 and then identified their counterparts in other representative strains of *Salmonella* and *E. coli* K12; in addition, we also profiled the degenerated forms of ACTAGT in the compared genomes based on their homology (genomic location and sequence similarity of the up-and down-stream DNA segments) to the ACTAGT sequences in *S. typhimurium* LT2 (Supplementary Table 1). Unlike most commonly used restriction enzymes that cleave a hexanucleotide sequence, such as EcoRI (GAATTC), BamHI (GGATCC), HinDIII (AAGCTT), etc., which have 751, 295 and 648 cleavage sites respectively on the genome of *S. typhimurium* LT2, SpeI (ACTAGT) has only 39 cleavage sites. Strains of other *Salmonella* subgroup I lineages and *S. bongori *strains have similar numbers, although the numbers in *S. arizonae* and *E. coli* were a little greater (around 80; Supplementary Table 2). Based on the numbers of shared homologous ACTAGT sequences among the bacteria, we constructed a dendrogram as described previously^15^ and compared it (Fig. 1A) to the core genome-based phylogenetic tree (Fig. 1B); the two trees are almost congruent with only some minor topological differences, indicating that the degeneration of ACTAGT is consistent with the divergence of the genomes by accumulating mutations independently in individual bacterial lineages.

### The most highly conserved ACTAGT sequences or their degenerated forms across the bacteria: many in intergenic regions

**Figure 2:**
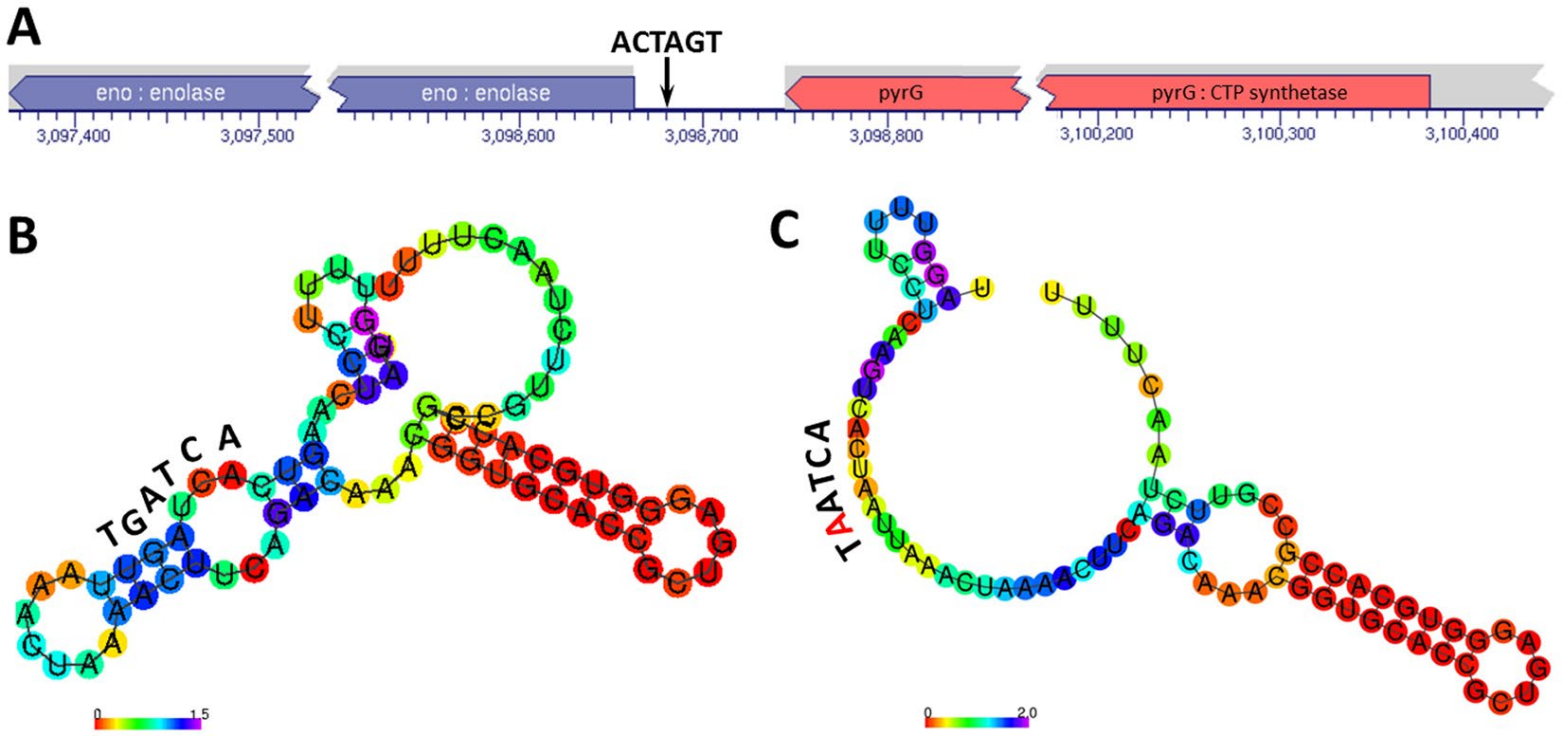
Analysis of the *eno-pyrG* intergenic sequence conserved between *Salmonella* and *E. coli*. (**A**) Genomic location; (**B**) Computer modeling of the secondary structure for the wild type hexanucleotide sequence ACTAGT; (**C**)Computer modeling of the secondary structure for a degenerated hexanucleotide sequence A*A*TAGT, in which a C → A transversion would result in the disruption of the stem-loop structure formed by ACTAGT.

The majority of the ACTAGT sequences in the genome of *S. typhimurium* LT2 had counterparts at homologous locations in the genomes of the other analyzed bacteria, either in the “wild type” form or in one of the degenerated forms, across different *Salmonella* subgroups (e.g., I, IIIa, V lineages) and in *E. coli* (See Supplementary Table 1). In the profiling, we ignored the ACTAGT sequences that are present within LTD segments in strains other than LT2. The most highly conserved ACTAGT sequences or their degenerated forms across different bacteria suggest their biological importance. Particularly interesting is the one between genes *eno* and *pyrG* (Fig. 2A), which is conserved in *Salmonella* (including subgroups I, IIIa and V), *E. coli* and many other enteric bacteria (Supplementary Table 3). Computer modeling demonstrates that this ACTAGT sequence forms part of a stable stem-loop structure (Fig. 2B) and that a base substitution would entirely disrupt this structure (Fig. 2C).

### Degenerated forms of ACTAGT as the evolutionary products of being free from natural selection

We identified many hexanucleotide sequences with one nucleotide difference from ACTAGT in the *Salmonella* genomes at homologous sites to the ACTAGT sequence in the *S. typhimurium* LT2 genome, which we hypothesized to be the products of degenerated ACTAGT as a result of being free from natural selection pressures. If so, a gradual degeneration process might be traceable among bacteria of different levels of relatedness; in addition, a single base variation may disrupt the functional structure of the ACTAGT-containing intergenic sequence. To test this hypothesis, we profiled the intergenic regions containing ACTAGT as well as its degenerated forms and conducted computer modeling. We focused on two ACTAGT-containing intergenic sequences representing different levels of conservation, including the one between genes *pyrH* and *frr* and the one between genes *eda* and *edd* in *S. typhimurium* LT2, with the former being conserved across different *Salmonella* lineages (subgroups I, IIIa and V) and the latter being conserved within only *Salmonella* subgroups I lineages (see their genomic locations and wild-type or degenerated form status in Supplementary Table 1).

**Figure 3:**
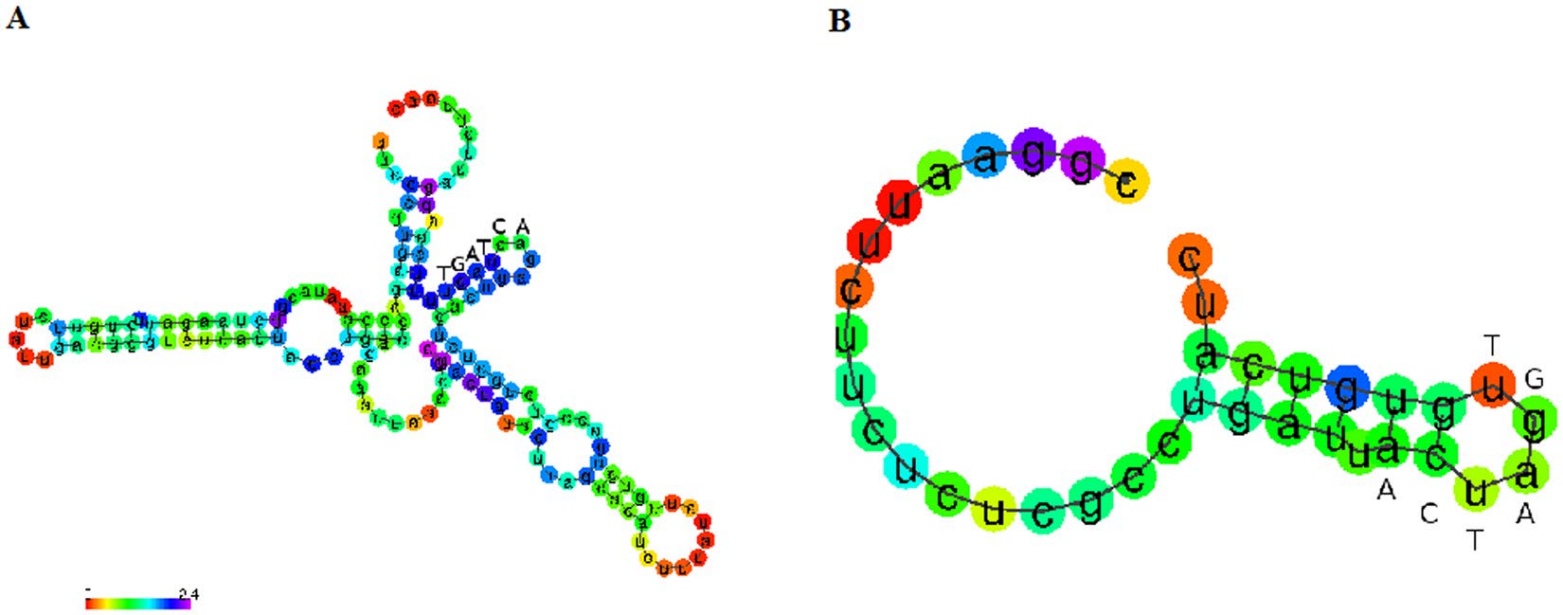
Prediction of secondary structures for intergenic sequences with different levels of evolutionary conservation. (**A**) The *pyrH*-*frr* intergenic sequence conserved across different *Salmonella* lineages (subgroups I, IIIa and V); (**B**) The *eda*-*edd* intergenic sequence conserved in *Salmonella* subgroup I but not in *Salmonella* subgroup IIIa or V. Both formed stable stem-loop structures but one-base mutation would produce structural modifications leading to one of the modeled degenerated forms in them with the stem-loop structure being disrupted or a thermally unstable stem-loop structure (see details in Supplementary Figures 1 and 2).

Both the *pyrH*-*frr* and *eda*-*edd* intergenic sequences formed thermally stable stem-loop structures (Fig. 3). As any long enough RNA sequence will form secondary structures, we limited the length of the intergenic sequences in the analysis and focused on the region surrounding the ACTAGT hexanucleotide to model all possible degenerated forms on each of them. Among the eighteen degenerated forms of ACTAGT modeled for the *pyrH*-*frr* intergenic region, three (*C*CTAGT, A*T*TAGT and AC*C*AGT) formed structures similar to that of ACTAGT but with lower thermal stability than that of ACTAGT (Supplementary Figure 1A,F,H). All analyzed *Salmonella* lineages of subgroups I, IIIa and V had the wild type ACTAGT sequence, suggesting its importance in function for these bacteria. Interestingly, *E. coli* K12 MG1655 had a degenerated form, AC*A*AGT, which retains a structure similar to that of ACTAGT but has lower thermal stability (Supplementary Figure 1G), suggesting the gradual loss of an ancestral function of this genomic segment in *E. coli* by mutation, instead of acquisition of this function multiple times by different *Salmonella* lineages. This finding provides important molecular entities for functional comparisons of ACTAGT and AC*A*AGT between *Salmonella* and *E. coli* regarding their biological differences. The ACTAGT sequence in the *eda*-*edd* intergenic region is wild type in all analyzed *Salmonella* subgroup I lineages but degenerated by two nucleotide substitutions in *Salmonella* subgroup IIIa and by three nucleotide substitutions in *Salmonella* subgroup V and *E. coli*(see Supplementary Table 1), suggesting entire function loss of the hexanucleotide in those bacteria, as judged by the fact that even one single nucleotide substitution would disrupt the stem-loop structure or make thermally much less stable structures (Supplementary Figure 2).

### Amino acid substitution caused by degeneration of ACTAGT: divergence to reduce the number of CTAG sequences?

Many ACTAGT sequences fall in predicted protein-coding regions and their degeneration may alter amino acid sequences, which provide excellent opportunities for evaluating how ACTAGT degeneration might influence functional molecules. For this, we profiled in-gene ACTAGT sequences or their degenerated forms, using the genome of *S. typhimurium* LT2 as a reference for the analysis of the ACTAGT sequence within genes in other bacteria (a genome of any other *Salmonella* strain studied here may be used as the reference). Most profiled degenerated forms involve synonymous nucleotide substitutions (Supplementary Table 4), such as T to C transitions (ACT → ACC or AGT− > AGC), C to T transitions (GAC → GAT or CTA → TTA), A to G transitions (CTA → CTG), A to T transversions (CTA → CTT). Such substitutions may reflect a tendency of the *Salmonella* genome to minimize the number of the CTAG tetranucleotide sequence^10, 28^, although we do not rule out the possibility that the substituting nucleotide might make the amino acid sequence more adaptive.

**Figure 4:**
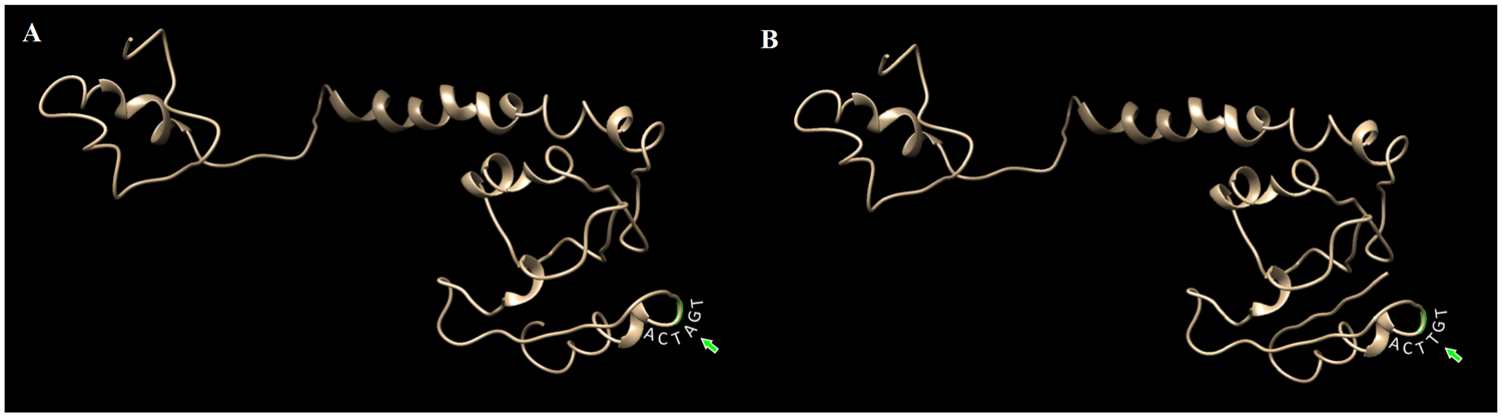
Prediction of possible structural changes for the N terminal portion of the resolvase encoded by gene *hin* in S*. typhimurium* LT2 when a leucine is substituted by serine due to an AGT → TGT transversion in *S. paratyphi* C. (**A**) Modeled structure in *S. typhimurium*; and (**B**) Modeled structure in *S. paratyphi* C. As the amino acid substitution takes place in a region connecting two helices, the secondary protein structure is not affected.

Notably, we also identified a transversion event, AGT → TGT, in the *hin* gene coding for a resolvase^29^, which changes serine to cysteine in *S. paratyphi* C and *S. choleraesuis* but not in other analyzed *Salmonella* subgroup I lineages. Computer modeling showed that this mutation falls in an N terminal segment that connects two helices so does not affect the secondary structure (Fig. 4), which is a special case reflecting a tendency of the *Salmonella* genome to minimize the number of the CTAG tetranucleotide sequence if the degeneration does not affect the functions of the molecule^9, 15^.

## Discussion

To gain a snapshot on the general patterns of genomic nucleotide composition optimization or amelioration in the process in which bacteria diverge into different niches such as distinct hosts (e.g., humans *vs* livestock or wild animals) or different tissues (causing systemic *vs* local infections), we analyzed a subset of the highly conserved short sequence CTAG among representative *Salmonella* pathogens by profiling the SpeI cleavage sequence ACTAGT in comparison with *E. coli* and other enteric bacteria. Since their divergence from *E. coli* over 100 million years ago^30–32^, *Salmonella* have evolved into over 2600 lineages, which have been treated as distinct serotypes and individual species^22^, serovars of a single or a couple of species^33–35^, or natural species delineated by genetic boundaries^21, 23^. When bacteria diverge from a common ancestor and migrate into different niches, the nascent lineages would gradually accumulate large arrays of genomic variations as demonstrated by the comparison of genetically similar but pathogenically distinct bacteria such as *S. paratyphi* C and *S. cholereasuis*, which have accumulated thousands of nucleotide differences between their genomes during a relatively short evolutionary period of time^7^. However, not much meaningful information has been extracted from such large amounts of nucleotide variations regarding, for example, their possible contributions to bacterial adaptation to various new environments.

We have focused on the identification of highly conserved short genomic sequences to reveal their general patterns of nucleotide composition changes between bacteria that have evolved in different directions to become local or systemic infection agents or to adapt to different hosts. We have recently reported profiling the tetranucleotide sequence CTAG to delineate the *Salmonella* bacteria into natural clusters^15^, which are equivalent to monophyletic *Salmonella* serotypes or species, and to identify some hitherto cryptic but potentially important intergenic sequences. However, we found in this study that the presence of CTAG may not necessarily indicate that the sequence containing it is still conserved, as the degeneration process may take some time even if the selection pressure to maintain it is no longer present. As shown in Supplementary Figures 1 and 2, computer modeling demonstrated that the mutation of a single base up- or down-stream to CTAG (i.e., the first or last base of the SpeI cleavage sequence ACTAGT) may entirely disrupt the stem-loop structure. Therefore, analysis of an extended sequence, e.g., one base each added to up- and down-stream of CTAG, such as the hexanucleotide SpeI cleavage sequence ACTAGT instead of CTAG, may in many cases add useful information to the CTAG profiles.

Profiling the SpeI cleavage sequence ACTAGT in this study revealed a general pattern of conservation loss in many sequences as a consequence of nucleotide variation: highly ordered structures would collapse when ACTAGT degenerates just by a single base. Interestingly, such sequence degeneration and structure disruption often take place at phylogenetic scales. For example, the *eno*-*pyrG* intergenic sequence (see Fig. 2A) is conserved in bacteria across a broad phylogenetic range including *Salmonella*, *E. coli* and many other genera (see Supplementary Table 3), but the *pyrH*-*frr* intergenic sequence is conserved in *Salmonella* (subgroups I, IIIa and V) but not *E. coli*, and the *eda*-*edd* intergenic sequence is conserved only in *Salmonella* subgroup I bacteria. Experimental studies for their functional comparisons may lead to novel insights into the molecular mechanisms for phylogenetic divergence and pathogenic evolution of bacteria.

In this study, we obtained new evidence showing a tendency of the *Salmonella* genomes to minimize the number of the CTAG tetranucleotide sequence where possible. Most in-gene degenerated forms of ACTAGT contain synonymous mutations (see Supplementary Table 1); in rare cases, however, the in-gene degenerated forms of ACTAGT did change the amino acid, such as the AGT → TGT transversion in the *hin* gene. Although this mutation led to the substitution of serine to cysteine, computer modeling showed that this mutation falls in a protein segment that connects two helices without affecting the secondary structure of the protein molecule (see Fig. 4). Based on this finding, we anticipate seeing relatively stable CTAG profiles in the genome of extant *Salmonella* and other enteric bacteria. Our findings suggest that, when some stochastic LTD insertions start new speciation processes in a bacterial branch, diverting its evolutionary direction and bringing it to a new niche, the nascent bacterial lineage would begin accumulating a new set of genomic variations and optimize the nucleotide composition by further reducing the number of ancestral or laterally acquired CTAG sequences. Once integrated into a bacterial genome, the newly acquired CTAG sequences may undergo the nucleotide composition refinement or amelioration process toward optimization to adapt to the host genomic environment.

## Electronic supplementary material

Supplementary information

STable 1

STable 2

STable 4
