# Supplementary material for: Differential degeneration of the ACTAGT sequence among Salmonella: a reflection of distinct nucleotide amelioration patterns during bacterial divergence
Source: Sci Rep. 2017 Sep 8;7:10985. doi: 10.1038/s41598-017-11226-9 (PMC5591236; doi:10.1038/s41598-017-11226-9)
Supplement: Supplementary file 1 — Supplementary information [file 41598_2017_11226_MOESM1_ESM.pdf]

Differential degeneration of the ACTAGT sequence among *Salmonella*: a reflection of distinct nucleotide amelioration patterns during bacterial divergence

Le Tang[1,2*#], Emilio Mastriani[1,2*], Yu-Jie Zhou[1-3*], Songling Zhu[1,2], Xin Fang[1,2], Yang-Peng Liu[1,2], Wei-Qiao Liu[4#], Yong-Guo Li[5], Randal N. Johnston[6], Zheng Guo[7], Gui-Rong Liu[1,2]**, Shu-Lin Liu[1-5]**

[1] Systemomics Center, College of Pharmacy, and Genomics Research Center (State-Province Key Laboratories of Biomedicine-Pharmaceutics of China), Harbin Medical University, Harbin, China;

[2]HMU-UCFM Centre for Infection and Genomics, Harbin Medical University, Harbin, China;

[3]Translational Medicine Research and Cooperation Center of Northern China, Heilongjiang Academy of Medical Sciences, Harbin, China;

[4]Department of Microbiology, Immunology and Infectious Diseases, University of Calgary, Calgary, Canada;

[5]Department of Infectious Diseases of First Affiliated Hospital, Harbin Medical University, Harbin, China;

[6]Department of Biochemistry and Molecular Biology, University of Calgary, Calgary, Canada;

[7]College of Bioinformatics Science and Technology, Harbin Medical University, Harbin, China

*These authors contributed equally to this work

#Current affiliations:

LT: School of Veterinary Medicine, University of Calgary, Calgary, Canada;

WQL: Department of Clinical Neurosciences, University of Calgary, Calgary, Canada, and Division of Neurology, Department of Medicine, University of British Columbia, Vancouver, Canada.

SUPPLEMENTARY INFORMATION

Computer modeling of degenerated forms of ACTAGT in the *pyrH-frr* intergenic region.

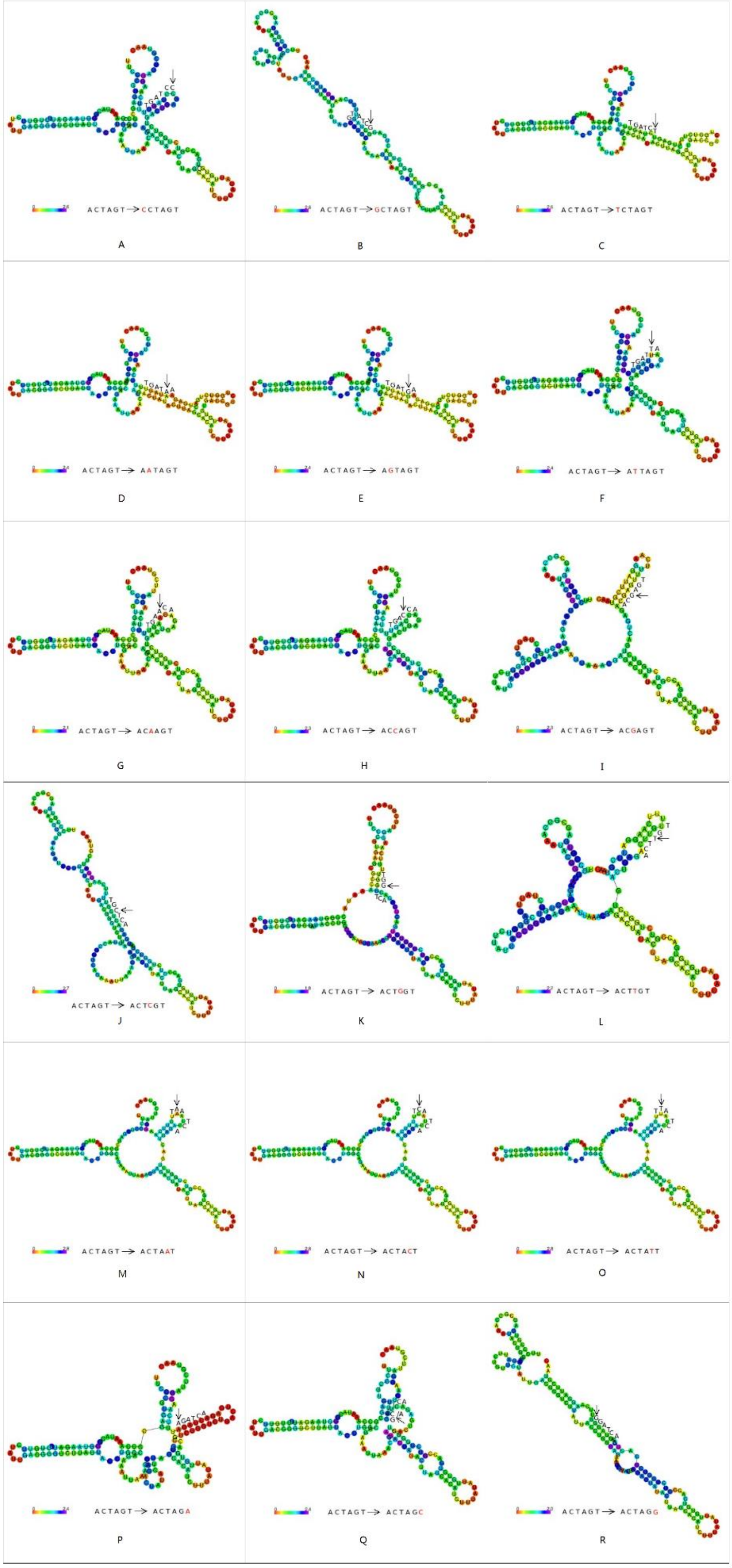

Supplementary Figure 1. RSS diagram of degenerated forms of ACTAGT in the *pyrH-frr* intergenic region. It shows the predicted structures using colors to emphasize the positional entropy inside the structure.

Computer modeling of degenerated forms of ACTAGT in the *eda-edd* intergenic region.

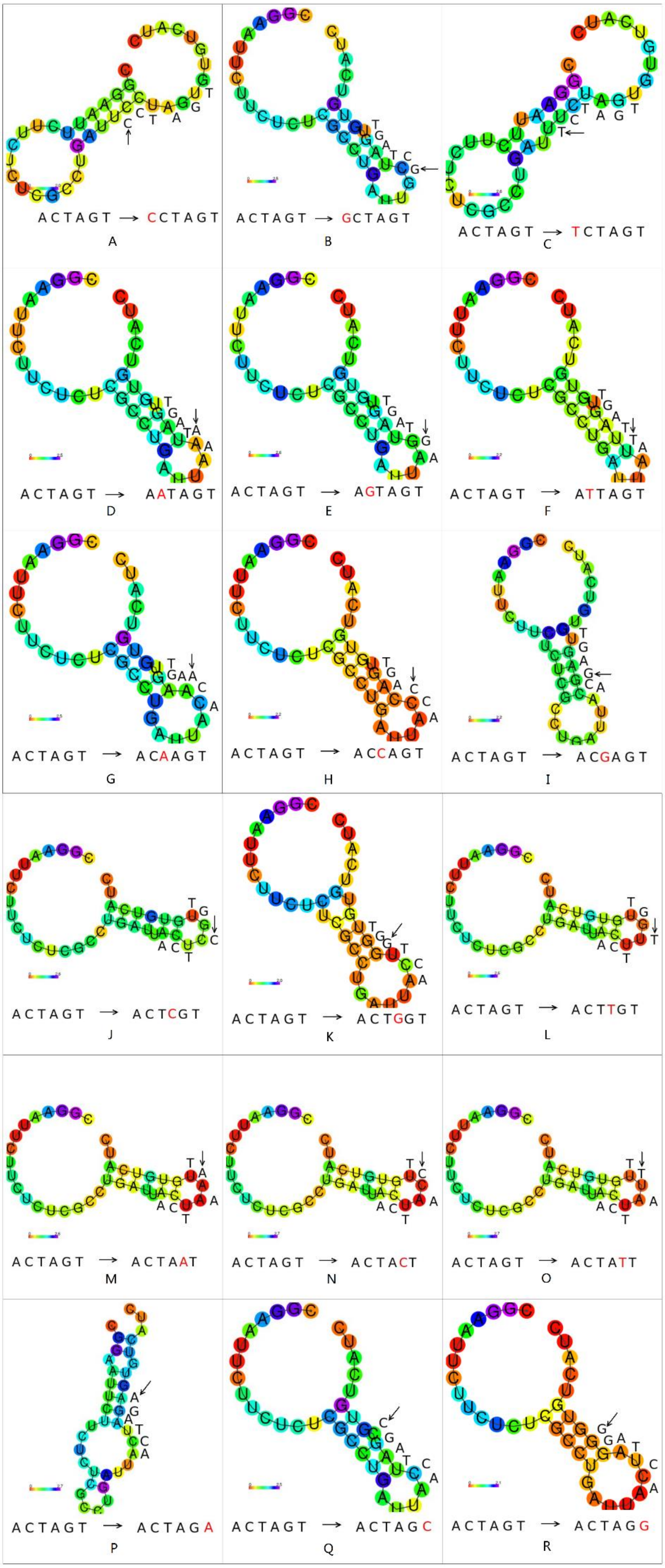

Supplementary Figure 2. RSS diagram of degenerated forms of ACTAGT in the *eda-edd* intergenic region. It shows the predicted structures using colors to emphasize the positional entropy inside the structure.

Supplementary Table 1. Profiles of the hexanucleotide sequence ACTAGT in *S. typhimurium* LT2 and its wild type or degenerated counterparts in other *Salmonella* strains and *E. coli* K12.

Please see the separate Excel documents because it is too large.

Supplementary Table 2. Numbers of the ACTAGT sequence in representative *Salmonella* strains and *E. coli* K12.

Please see the separate Excel documents because it is too large.

Supplementary Table 3. Conservation of the *eno-pyeG* intergenic region containing the ACTAGT sequence among the enteric bacteria

Bacterial genome	Max score	Total score	Query cover	E value	Ident	Accession
Salmonella enterica subsp. enterica serovar Paratyphi A str. ATCC 9150, complete genome	224	224	100%	1.00E-56	100%	NC_006511.1
Salmonella bongori NCTC 12419, culture collection SGSC SARC11, complete genome	224	224	100%	1.00E-56	100%	NC_015761.1
Salmonella enterica subsp. enterica serovar Typhi str. CT18, complete genome	224	224	100%	1.00E-56	100%	NC_003198.1
Salmonella enterica subsp. enterica serovar Typhimurium str. LT2 chromosome, complete genome	224	224	100%	1.00E-56	100%	NC_003197.1
Klebsiella oxytoca KCTC 1686, complete genome	161	161	100%	1.00E-37	91%	NC_016612.1
Raoultella ornithinolytica B6, complete genome	156	156	100%	5.00E-36	90%	NC_021066.1
Klebsiella pneumoniae subsp. pneumoniae HS11286 chromosome, complete genome	156	156	100%	5.00E-36	90%	NC_016845.1
Klebsiella variicola At-22, complete genome	156	156	100%	5.00E-36	90%	NC_013850.1
Klebsiella pneumoniae subsp. pneumoniae MGH 78578, complete sequence	156	156	100%	5.00E-36	90%	NC_009648.1
Enterobacter aerogenes KCTC 2190 chromosome, complete genome	150	150	100%	2.00E-34	89%	NC_015663.1
Escherichia coli str. K-12 substr. MG1655, complete genome	141	141	72%	1.00E-31	95%	NC_000913.3
Escherichia coli O104:H4 str. 2011C-3493 chromosome, complete genome	141	141	72%	1.00E-31	95%	NC_018658.1
Escherichia coli O83:H1 str. NRG 857C chromosome, complete genome	141	141	72%	1.00E-31	95%	NC_017634.1

Shigella flexneri 2a str. 301 chromosome, complete genome	141	141	72%	1.00E-31	95%	NC_004337.2
Escherichia coli UMN026 chromosome, complete genome	141	141	72%	1.00E-31	95%	NC_011751.1
Escherichia coli IAI39 chromosome, complete genome	141	141	72%	1.00E-31	95%	NC_011750.1
Shigella boydii Sb227, complete genome	141	141	72%	1.00E-31	95%	NC_007613.1
Shigella dysenteriae Sd197 chromosome, complete genome	141	141	72%	1.00E-31	95%	NC_007606.1
Shigella sonnei Ss046, complete genome	141	141	72%	1.00E-31	95%	NC_007384.1
Escherichia coli O157:H7 str. Sakai chromosome, complete genome	141	141	72%	1.00E-31	95%	NC_002695.1
Serratia liquefaciens ATCC 27592, complete genome	139	139	72%	5.00E-31	95%	NC_021741.1
Serratia plymuthica 4Rx13, complete genome	139	139	72%	5.00E-31	95%	NC_021591.1
Shimwellia blattae DSM 4481 = NBRC 105725, complete genome	139	139	71%	5.00E-31	95%	NC_017910.1
Serratia plymuthica AS9, complete genome	139	139	72%	5.00E-31	95%	NC_015567.1
Serratia sp. AS12, complete genome	139	139	72%	5.00E-31	95%	NC_015566.1
Citrobacter rodentium ICC168, complete genome	139	139	72%	5.00E-31	95%	NC_013716.1
Serratia proteamaculans 568, complete genome	139	139	72%	5.00E-31	95%	NC_009832.1
Enterobacter asburiae LF7a, complete genome	134	134	72%	2.00E-29	94%	NC_015968.1
Cronobacter turicensis z3032 complete genome	134	134	72%	2.00E-29	94%	NC_013282.2
Enterobacter lignolyticus SCF1, complete genome	134	134	72%	2.00E-29	94%	NC_014618.1
Enterobacter cloacae subsp. cloacae ATCC 13047 chromosome, complete genome	134	134	72%	2.00E-29	94%	NC_014121.1

Cronobacter sakazakii ATCC BAA-894, complete genome	134	134	72%	2.00E-29	94%	NC_009778.1
Enterobacter sp. 638, complete genome	128	128	72%	1.00E-27	93%	NC_009436.1
Yersinia pseudotuberculosis PB1/+, complete genome	122	122	70%	5.00E-26	93%	NC_010634.1
Yersinia enterocolitica subsp. enterocolitica 8081 chromosome, complete genome	122	122	70%	5.00E-26	93%	NC_008800.1
Yersinia pseudotuberculosis IP32953 genome, complete sequence	122	122	70%	5.00E-26	93%	NC_006155.1
Yersinia pestis CO92 chromosome, complete genome	122	122	70%	5.00E-26	93%	NC_003143.1
Yersinia pestis KIM10+, complete genome	122	122	70%	5.00E-26	93%	NC_004088.1
Edwardsiella piscicida C07-087, complete genome	121	121	69%	2.00E-25	93%	NC_020796.1
Edwardsiella tarda EIB202, complete genome	121	121	69%	2.00E-25	93%	NC_013508.1
Edwardsiella ictaluri 93-146, complete genome	115	115	69%	8.00E-24	92%	NC_012779.2
Rahnella aquatilis CIP 78.65 = ATCC 33071, complete genome	111	111	66%	1.00E-22	93%	NC_016818.1
Erwinia sp. Ejp617, complete genome	110	110	70%	4.00E-22	91%	NC_017445.1
Erwinia pyrifoliae strain Ep1/96 complete chromosome	110	110	70%	4.00E-22	91%	NC_012214.1
Erwinia amylovora ATCC 49946 chromosomal sequence	108	108	71%	1.00E-21	90%	NC_013971.1
Erwinia billingiae strain Eb661 complete chromosome	104	104	72%	2.00E-20	89%	NC_014306.1
Yersinia pestis Pestoides F, complete genome	104	104	59%	2.00E-20	93%	NC_009381.1
Erwinia tasmaniensis strain ET1/99 complete chromosome	99	99	70%	8.00E-19	88%	NC_010694.1
Providencia sneebia DSM 19967 chromosome, whole genome shotgun sequence	97.1	97.1	53%	3.00E-18	94%	NZ_CM001773.1
Photorhabdus asymbiotica ATCC43949 complete genome	97.1	97.1	56%	3.00E-18	93%	NC_012962.1

Photorhabdus luminescens subsp. laumondii TTO1 complete genome	97.1	97.1	56%	3.00E-18	93%	NC_005126.1
Xenorhabdus nematophila ATCC 19061 chromosome, complete genome	89.8	89.8	57%	5.00E-16	90%	NC_014228.1
Xenorhabdus bovienii SS-2004 chromosome, complete genome	86.1	86.1	56%	6.00E-15	90%	NC_013892.1
Actinobacillus succinogenes 130Z, complete genome	69.4	69.4	45%	6.00E-10	89%	NC_009655.1
Mannheimia succiniciproducens MBEL55E, complete genome	60.2	60.2	36%	4.00E-07	91%	NC_006300.1

Supplementary Table 4. In-gene ACTAGT sequences in *S. typhimurium* LT2 and their wild type or degenerated counterparts in other *Salmonella* lineages.

Please see the separate Excel documents because it is too large.
